# Fluralaner 5.46% (w/w) flavored chewable tablet (Bravecto^®^ 1-Month) is effective for treatment of canine generalized demodicosis

**DOI:** 10.1186/s13071-022-05213-x

**Published:** 2022-03-12

**Authors:** Nadja Rohdich, Leon Meyer, Frank Guerino

**Affiliations:** 1 MSD Animal Health Innovation GmbH, Zur Propstei, 55270 Schwabenheim, Germany; 2 Clinvet International (Pty) Ltd, Uitzich Road, Bainsvlei, Bloemfountein, South Africa; 3 Merck Animal Health, 2 Giralda Farms, Madison, NJ 07940 USA

**Keywords:** Bravecto^®^, Canine, *Demodex*, Generalized demodicosis, Imidacloprid-moxidectin, Fluralaner

## Abstract

**Background:**

Orally administered fluralaner (13.64% w/w) is effective for treating canine generalized demodicosis. A study was initiated to assess the efficacy of a novel 5.46% w/w fluralaner chewable tablet formulation for monthly administration in the treatment of this disease.

**Methods:**

Client-owned dogs diagnosed with generalized demodicosis were acclimatized to laboratory conditions and randomized to receive either orally administered fluralaner (Bravecto^®^ 1-Month) (10.0 to 14.4 mg/kg body weight) (*n* = 8) or topical imidacloprid-moxidectin (Advocate^®^ for dogs, Elanco) applied per label on days 0, 28, and 56 (*n* = 8), or more frequently for ongoing severe demodicosis. On days −2, 28, 56, and 84, deep skin scrapings were taken from five sites on each dog for mite identification and counting, and semiquantitative clinical assessments of generalized demodicosis were recorded. Primary efficacy was based upon arithmetic mean mite count reductions relative to pre-treatment.

**Results:**

By day 28, mean pre-treatment mite counts, > 600 in both groups, were significantly reduced by 99.7% and 89.5% (both *P* < 0.001) in the fluralaner and imidacloprid-moxidectin groups, respectively. Parasitological cure (100% reduction in mite counts on days 56 and 84) was achieved in all fluralaner-treated dogs (100%) and in two imidacloprid-moxidectin-treated dogs (25%). In the imidacloprid-moxidectin group, the reduction in mean mite counts was 89.5% (day 28), 94.4% (day 56), and 97.5% (day 84). All study dogs were free of crusts on days 56 and 84. Scales resolved by day 84 in all fluralaner-treated dogs and in three imidacloprid-moxidectin-treated dogs. All fluralaner-treated dogs and five imidacloprid-moxidectin-treated dogs had > 90% hair regrowth on day 84.

**Conclusion:**

Three consecutive monthly orally administered treatments with fluralaner (5.46% w/w) flavored chewable tablets (minimum dose rate 10 mg/kg body weight) eliminated *Demodex canis* mites from dogs diagnosed with generalized demodicosis.

**Graphical Abstract:**

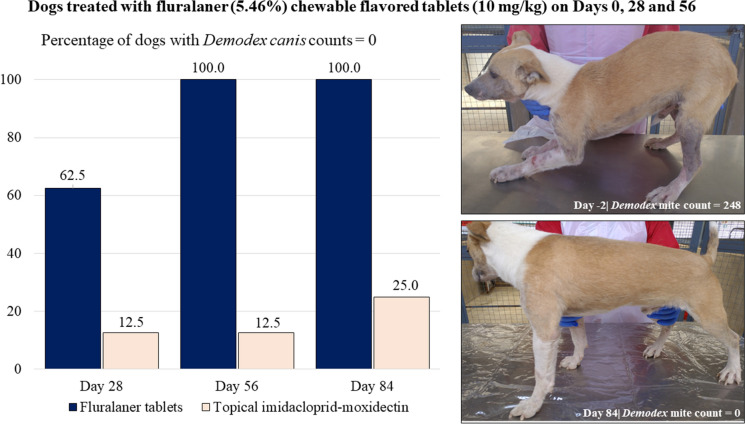

## Background

Canine demodicosis is an inflammatory parasitic skin disease characterized by an excessive multiplication of *Demodex canis* mites within the pilosebaceous glands [1]. Localized demodicosis is generally a benign disease, with most cases resolving spontaneously within 6 to 8 weeks of the appearance of clinical signs [2]. The disease is defined as generalized when five or more areas of the body are affected, pododemodicosis is observed on at least two paws, or an entire body region is involved [3, 4]. Demodicosis is also categorized as either juvenile- (dogs up to 18 months of age) or adult-onset (dogs generally older than 4 years of age with no previous history of disease) [4]. Generalized demodicosis is unlikely to resolve without therapy and has long posed a challenge to veterinarians in implementing successful treatment [2, 4–6]. Until recently, one product, a topically applied combination formulation of imidacloprid and moxidectin, was registered and available in Europe for the treatment of canine generalized demodicosis. However, even with weekly retreatment, efficacy has been shown to be incomplete [5, 7–9]. The discovery and registration of the isoxazoline family of compounds provided a breakthrough in the treatment of this complex disease [7–10].

The isoxazoline compound fluralaner was first available in 2014 as a flavored chewable tablet formulation to provide up to 12 weeks’ efficacy against canine flea and common tick infestations, and is now also approved for the treatment of sarcoptic and demodectic mange infestations [11]. In 2016 a topical formulation of fluralaner was approved for use in cats as well as dogs to provide the same efficacy and duration as the original oral formulation for the treatment and control of fleas and ticks, as well as sarcoptic and demodectic mite infestations in dogs and ear mite (*Otodectes cynotis*) infestations in cats [11]. More recently, a novel 5.46% (w/w) fluralaner chewable tablet formulation was developed for monthly administration at a minimum dose rate of 10 mg/kg to control canine flea and tick infestations [12]. This study was conducted to evaluate the efficacy of the new tablet formulation in the treatment of canine generalized demodicosis when administered three times at monthly intervals.

## Methods

This study was conducted in accordance with the VICH GL9 (June 2000) guideline on Good Clinical Practice [13], and the 2010 Guideline on Statistical Principles for Veterinary Clinical Trials [14]. Ethical approval was obtained by the Clinvet Institutional Animal Care and Use Committee before beginning the study, and all owners signed informed consent forms after receiving written and verbal details of the study procedures and products to be administered.

### Dogs and management

To qualify for the study, dogs were required to be clinically healthy, except for showing signs associated with generalized demodicosis. The demodicosis signs required for inclusion in the study were pododemodicosis involving two or more paws, or one or more of erythema, hair loss, comedones, follicular casts, scales, or crusts affecting either an entire body region or localized to more than five body areas. Dogs were excluded if they had received glucocorticoid therapy or any ectoparasiticide or endectocide within 12 weeks prior to day 0, if known to be pregnant, or if fractious to the point of posing a danger to themselves or facility personnel.

Adult dogs of mixed breeds and unknown ages were sourced from rural areas with limited access to animal health care. Following owner consent, 16 suitable dogs were transferred to the study site to undergo a 7-day acclimatization period, during which daily health observations were made, with clinical examinations and body weight measurements completed on days −7 and −2. Starting on day −7 all dogs received an appropriate antibiotic (Convenia^®^, cefovecin, Zoetis) as a treatment for pyoderma. As standard practice for the laboratory, the dogs also received a probiotic (Protexin^®^ Soluble, Kyron Laboratories) from day −7 until the end of the study on day 84 to minimize any risk of gastrointestinal disturbance arising from the change to formulated diet and cephalosporin-related side effects. Demodicosis was confirmed by identification of *D. canis* mites from deep skin scrapings completed on day −2.

During the study, dogs were kept individually in concrete-floored pens under strict quarantine conditions. No physical contact between dogs was possible, but dogs had visual and auditory contact with conspecifics. The dog pens consisted of a 1.7 m × 0.7 m enclosed sleeping area, with panel heaters, plastic beds/rubber mats, and an outside run of 1.7 m × 3.0 m. Standard commercially available diets that met the nutritional requirements of the dogs were fed at the recommended rates, and water was provided in stainless steel bowls and replenished at least twice daily. Food was removed in the afternoon prior to skin biopsies to facilitate anesthesia for biopsy procedures. At least one toy/chew was available to each dog (replenished weekly). A roof covering the kennels prevented exposure to rain and dogs were held in ambient temperatures with natural lighting.

### Study treatments

On treatment days, dogs consumed up to half of their daily food ration within approximately 20 min before treatment, after which they were offered the remainder of the ration so that all were in the fed state at the time of treatment. Dogs were ranked according to live mite counts taken on day −2 and randomized to a treatment group. Dogs allocated to group 1 were treated with a new formulation of fluralaner (5.46% w/w flavored chewable tablets) (Bravecto^®^ 1-Month) on days 0, 28, and 56 at a minimum dose rate of 10 mg/kg (actual dose rates 10.0 to 14.4 mg/kg). Group 2 dogs were treated with a commercially available topical formulation containing imidacloprid (100 mg/ml) and moxidectin (25 mg/ml) (Advocate^®^) administered according to label directions (10 mg imidacloprid/kg, 2.5 mg moxidectin/kg). Treatments with imidacloprid-moxidectin were applied directly to the skin at one spot between the shoulder blades. Dogs were restrained for approximately 1 min following topical administration and any drip-off was re-applied. Although scheduled for applications on days 0, 28, and 56, the frequency of treatments with this topical product could be increased to once weekly if the blinded attending veterinarian diagnosed the demodicosis as being severe. Appropriate dosing for study treatments was calculated according to each dog’s body weight, assessed on days −2, 27, and 56. On day −2, the weight of dogs in group 1 ranged from 9.7 to 17.6 kg (median 13.0 kg) and in group 2 from 7.1 to 16.4 kg (median 9.0 kg). To prevent unmasking during assessments, all dogs in the fluralaner group received saline solution topically during the treatment process. If any dogs in the imidacloprid-moxidectin group required weekly treatment, all dogs in both treatment groups, excluding those receiving the active topical product, were treated topically with saline solution on the same days to maintain masking of study personnel. All dogs were observed hourly for up to 4 h after treatment and full clinical examinations were completed at 2-week intervals from days 14 to 84. No additional treatments with potential miticidal activity were used during the study on the dogs or their environment.

### Mite counts, clinical assessments, and skin biopsies

On days −2, 28, 56, and 84, deep scrapings (~ 4 cm^2^) were taken from five affected sites on each study dog. Scraping sites were recorded and these sites or sites of new lesions were scraped at each subsequent examination. To ensure the collection of any mites, scrapings were made with a blade so that capillary oozing occurred. Subsequent deep scrapings were made from the same sites or from sites of new lesions. Each scraping was transferred to a uniquely identified microscope slide containing mineral oil and examined under a stereomicroscope to identify and count live *D. canis* mites.

Clinical signs were observed and recorded by veterinary investigators with previous experience in many studies of canine demodicosis. Casts, scales, crusts, erythema, and pyoderma were assessed on the days on which scrapings were made and recorded as present or absent. Scoring was recorded for the degree of alopecia (1 = slight thinning of hair; 2 = conspicuous hair loss; 3 = no hair) and a semiquantitative estimate of hair regrowth compared to the pre-treatment assessment was also completed (1 = 0 to 50%; 2 =  > 50% to ≤ 90%; 3 =  > 90%). The overall severity of demodicosis (mild, moderate, severe) was evaluated by the blinded veterinarian investigator.

On days −7 and 27, biopsies from affected skin areas were taken from each dog under general anesthesia (propofol 1.0%). Any dogs with clinical signs of pyoderma at the day 27 skin biopsy examination were to be treated with an appropriate antibiotic (Convenia^®^), administered according to label. Antibiotic therapy was continued until receipt of the biopsy results, and if evidence of active infection was present the antibiotic treatment was continued in affected dogs for another 4 weeks. From day 0 until the end of the study all clinical assessments and mite counts were completed by qualified personnel blinded to the treatment group.

### Statistical analysis

The European Committee for Medicinal Veterinary Products (CVMP) guideline for evaluation of the efficacy of anti-parasitic substances for the treatment and prevention of tick and flea infestations in cats and dogs states that at least six animals should be used per group [15]. For this study, the guideline minimum was exceeded with two additional dogs included in each treatment group.

Primary efficacy was based upon the percent reduction in arithmetic mean mite counts relative to pre-treatment counts.

$$ {\text{Reduction}}\,{\mkern 1mu} \left[ \%  \right] = 100{\mkern 1mu} \, \times \,{\mkern 1mu} \frac{{{\text{Mean}}_{{{\text{pre}}}}  - {\text{Mean}}_{{{\text{post}}}} }}{{{\text{Mean}}_{{{\text{pre}}}} }} $$

where Mean_pre_ was the day −2 mite counts and Mean_post_ was derived from counts made on days 28, 56, and 84.

Geometric mean mite counts were calculated using logarithm transformed counts (count + 1) with 1 subsequently subtracted and analyzed using a linear mixed model. The fixed factor in the model was the time point. An unadjusted comparison of the pre-treatment versus post-treatment mite counts was performed using a two-tailed test where the level of significance was set to α = 0.05.

The parasitological cure rate was defined as the percentage of treated dogs having two consecutive negative scrapings on days 56 and 84. Non-inferiority and superiority of the proportion of cured fluralaner-treated dogs in comparison to the proportion of cured dogs in the imidacloprid-moxidectin group were investigated using the Farrington–Manning test of non-inferiority for the risk difference [16] with a level of significance of α = 0.025 and a tolerated difference of δ = 0.15. Both *P*-value and lower 97.5% one-sided confidence limits were calculated. If the lower confidence limit was above −0.15, non-inferiority was concluded. If the lower confidence limit was above 0, superiority was concluded.

## Results

One dog was removed from the fluralaner-treated group during the study because pregnancy was detected on day 28 and the study protocol excluded pregnant dogs, leaving seven dogs for assessment in the fluralaner group. The mean body weight of all dogs in both groups increased throughout the study. No treatment-related adverse events were recorded in either group throughout the study period.

### Mite counts

All study dogs met the inclusion criteria for generalized demodicosis, with mites observed in deep scrapings and accompanying clinical signs.

**Fig. 1:**
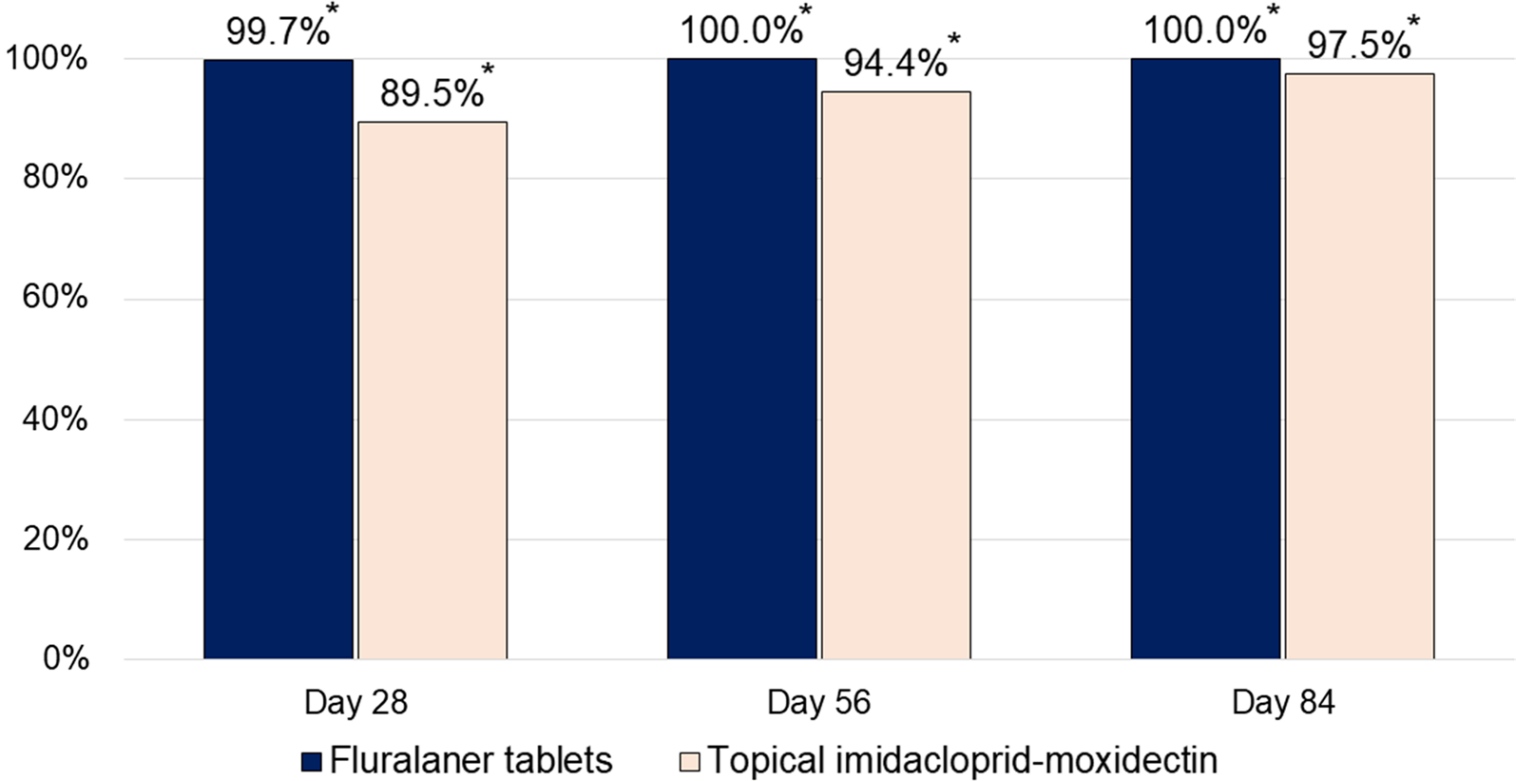
Percentage reduction in *Demodex canis* arithmetic mean mite counts in fluralaner- or imidacloprid-moxidectin-treated dogs with generalized demodicosis

Arithmetic mean pre-treatment mite counts in each group exceeded 600, and by day 28 mite counts were reduced by 99.7% in the fluralaner group and 89.5% in the imidacloprid-moxidectin group (Table 1; Fig. 1). Efficacy was 100% in the fluralaner group (days 56 and 84) and in the imidacloprid-moxidectin group efficacy was 94.4% (day 56) and 97.5% (day 84). Because of the continuing severity of the clinical signs of demodicosis, dogs in the imidacloprid-moxidectin group received repeated weekly treatments: two dogs needed 12 treatments (i.e., each week of the study), three dogs needed nine treatments and three needed six treatments. One *Sarcoptes scabiei* mite was observed in one dog in the imidacloprid-moxidectin group prior to treatment, but no *Sarcoptes* spp. mites were seen at any post-treatment assessment.

**Table 1:** Results from counts of *Demodex canis* mites obtained from deep skin scrapings

		Fluralaner tablets	Topical imidacloprid-moxidectin
Day −2	Arithmetic mean (SD)	639.9 (589.9)	608.5 (459.5)
	Median	507.5	459.0
	Range	19‒1764	90‒1314
	Geometric mean	347.5	432.3
Day 28	Arithmetic mean (SD)	2.0 (2.8)	63.9 (118.3)
	Median	0.0	17.0
	Range	0‒6	0–351
	*P*-value (vs day −2)	< 0.0005*	< 0.0001*
	Test statistics	*t*_(19)_ = 4.18	*t*_(21)_ = 5.05
	Geometric mean	1.0	17.2
Day 56	Arithmetic mean (SD)	0 (0.0)	34.1 (70.3)
	Median	0.0	11.0
	Range	0‒0	0‒207
	*P*-value (vs day −2)	0.0007*	< 0.0001*
	Test statistics	*t*_(19)_ = 4.05	*t*_(21)_ = 5.33
	Geometric mean	0.0	8.5
Day 84	Arithmetic mean (SD)	0 (0.0)	15.0 (22.8)
	Median	0.0	3.5
	Range	0‒0	0‒65
	*P*-value (vs day −2)	0.0007*	< 0.0001*
	Test statistics	*t*_(19)_ =4.05	*t*_(21)_ = 5.51
	Geometric mean	0.0	5.1

SD standard deviation

*Statistically significant

**Fig. 2:**
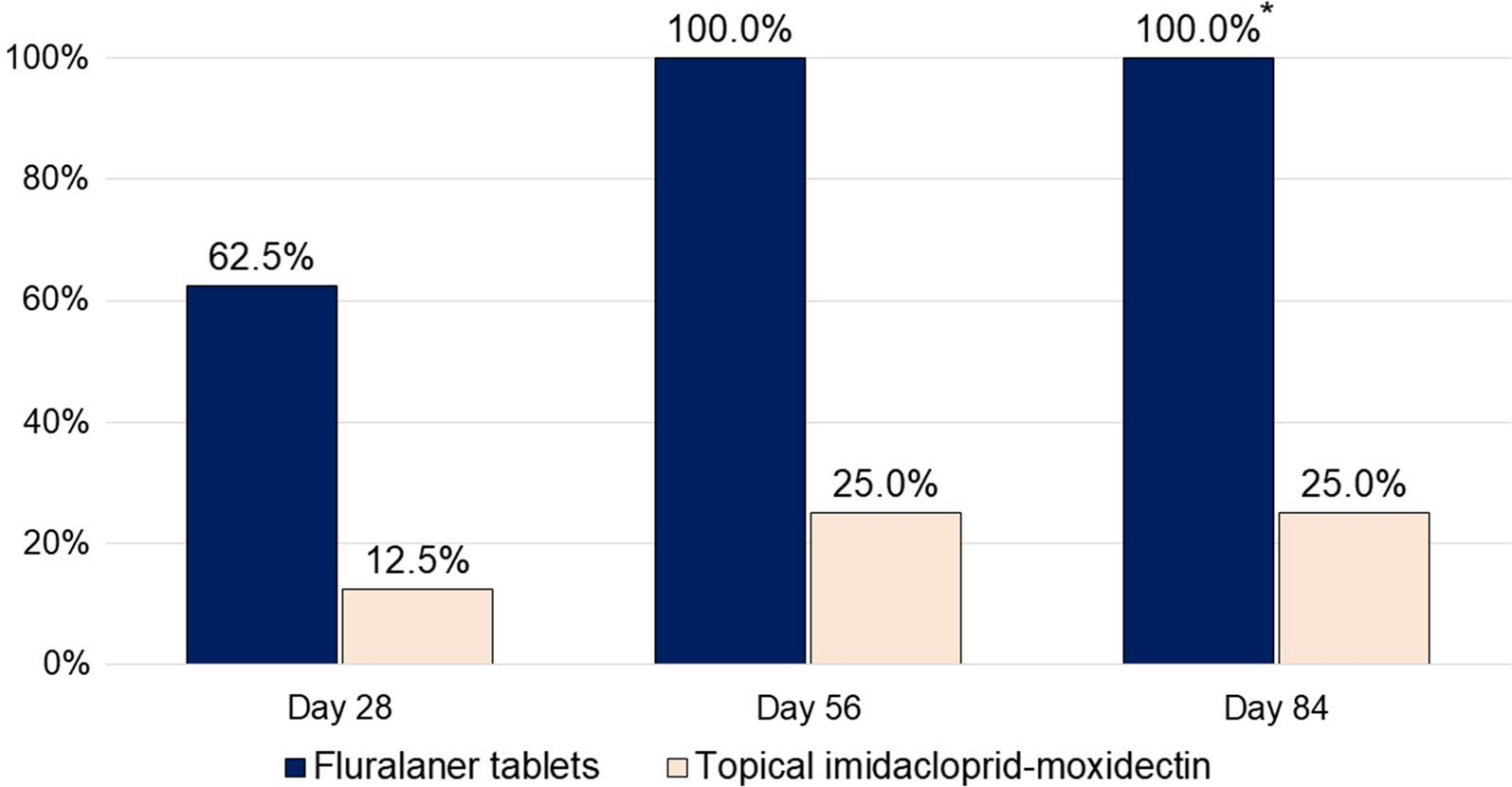
Percentage of dogs in each group free from *Demodex canis* mites; *between-group difference significant (*P* = 0.0002)

**Fig. 3:**
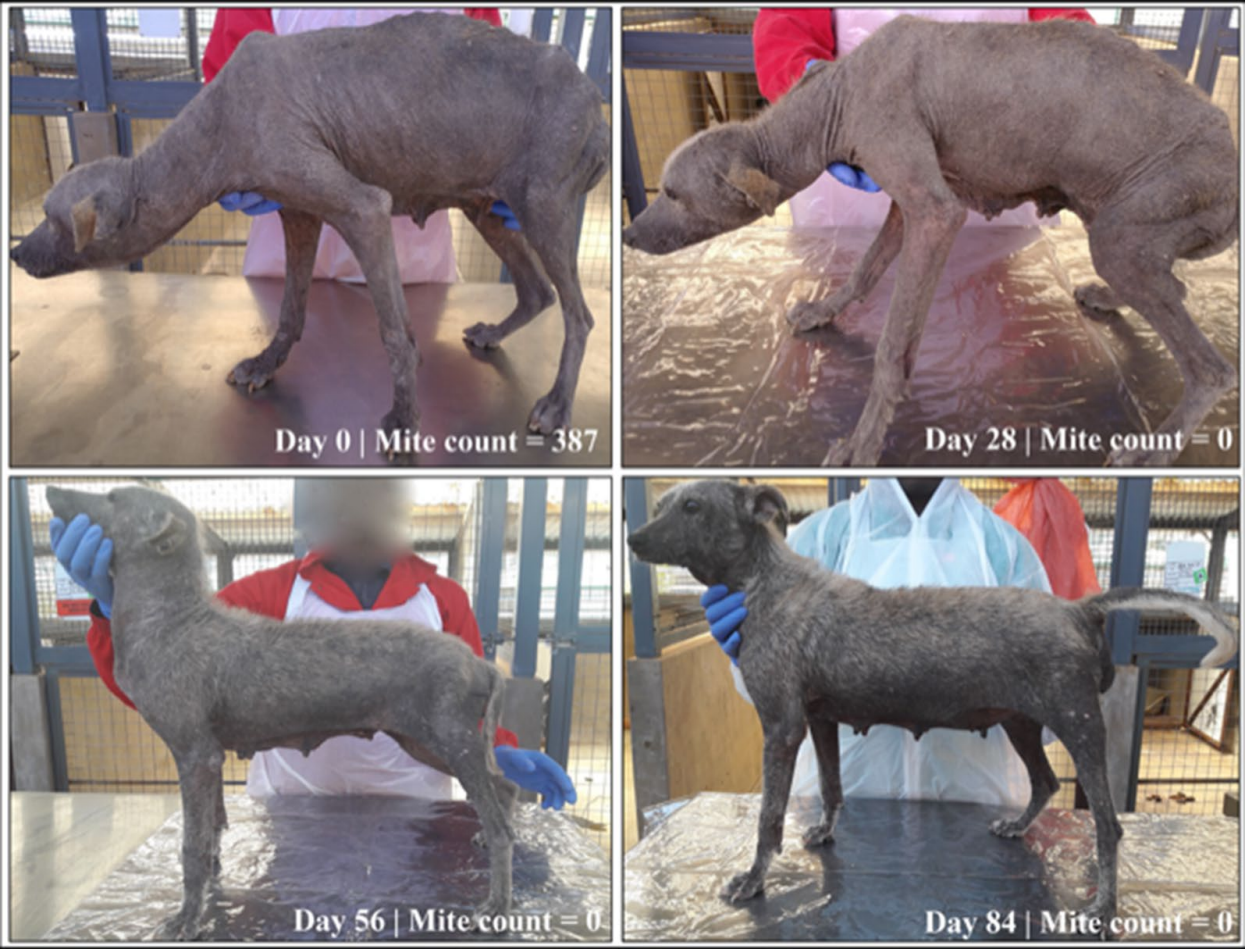
Dog in the imidacloprid-moxidectin group with a mite count = 0 on days 28, 56, and 84 but with continuing signs of demodicosis rated as severe

Mite counts from five of eight fluralaner-treated dogs were zero in scrapings taken immediately prior to the treatment on day 28 (Fig. 2). All seven fluralaner-treated dogs were negative for mites on days 56 and 84, meeting the definition of parasitological cure (two consecutive monthly assessments with zero mites). Parasitological cure was recorded in two of the eight imidacloprid-moxidectin-treated dogs. One imidacloprid-moxidectin-treated dog that received 12 weekly treatment applications had mite counts reduced to zero at the first post-treatment assessment on day 28 and then at each subsequent assessment with no recorded improvement in clinical signs. This was the only study dog with a severe demodicosis classification on day 84 (Fig. 3). The number of parasitologically cured dogs in the fluralaner group (100%) was significantly non-inferior (*P* = 0.0002, *Z* = 3.5525, *df* = 1) compared to the number in the imidacloprid-moxidectin group (25%). With the lower 97.5% confidence limit at 0.253, the superiority of fluralaner over imidacloprid-moxidectin could be concluded.

### Dermatological assessments

**Fig. 4:**
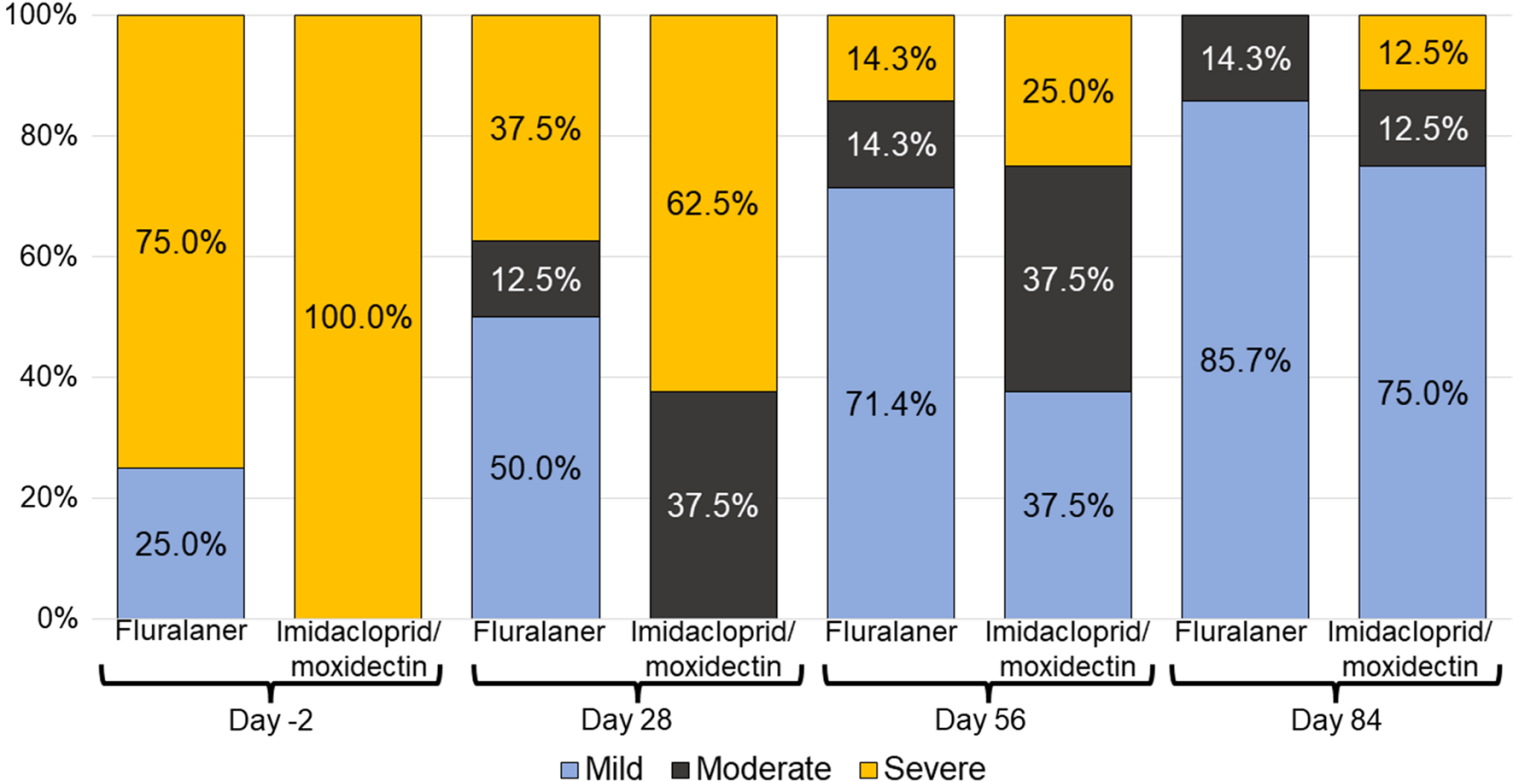
Demodicosis lesion severity in fluralaner- or imidacloprid-moxidectin-treated dogs on post-treatment days 28, 56, and 84

**Fig. 5:**
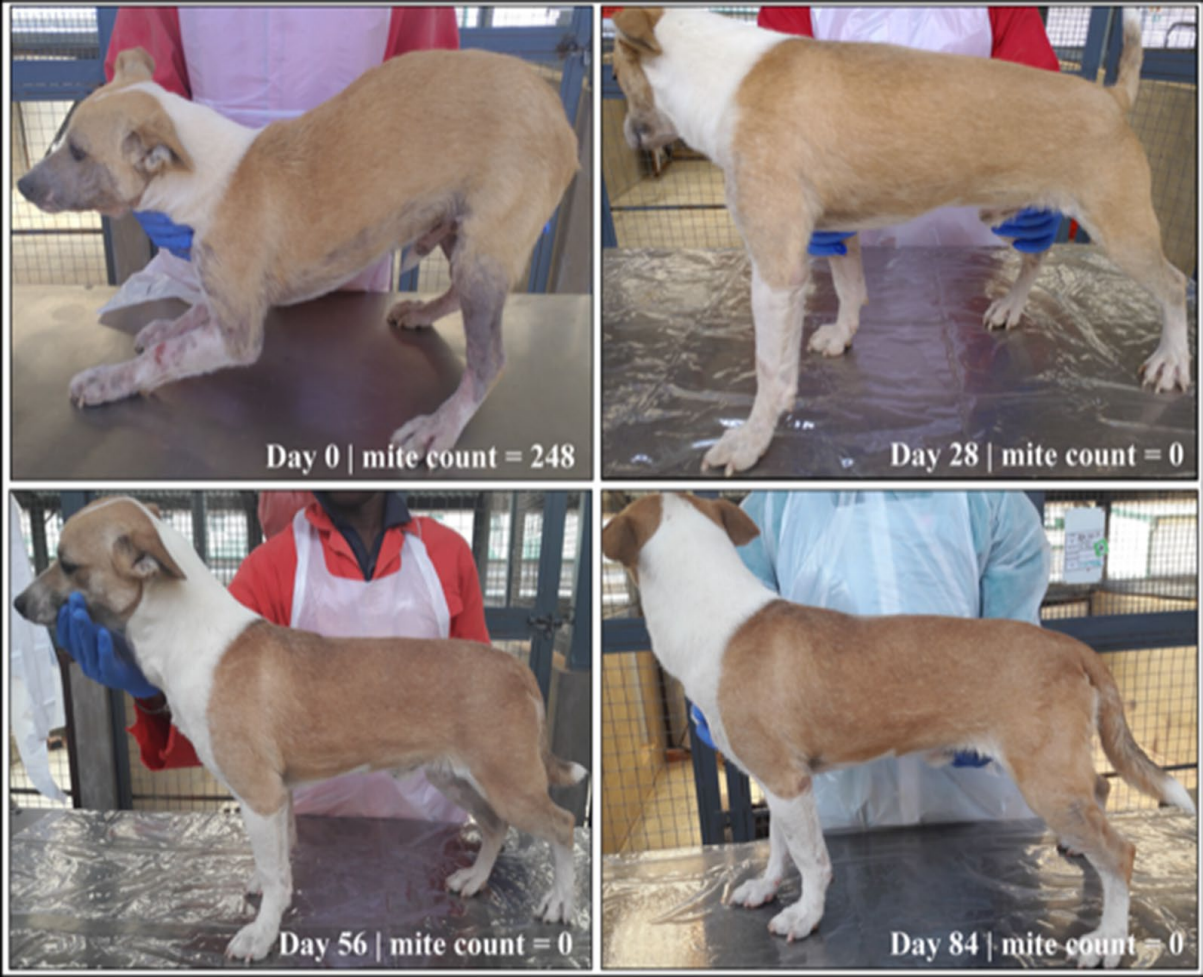
Pre- and post-treatment picture of a dog in the fluralaner group graded as having severe demodicosis lesions on day −2

All dogs in the imidacloprid-moxidectin group and five dogs in the fluralaner group had severe generalized demodicosis pre-treatment on day −2 (Fig. 4). All dogs in both groups were free of crusts at examination on days 56 and 84 (Table 2). Scales resolved by day 84 in all fluralaner group dogs and in five of eight imidacloprid-moxidectin group dogs. No casts, erythematous papules, or pyoderma were observed on any dog in either study group on day 0 or at any subsequent assessment (a protocol deviation was recorded to address the precautionary antibiotic treatment that was administered in the absence of diagnosed pyoderma). Marked hair regrowth was observed in all study dogs at day 84. All fluralaner-treated dogs had > 90% hair regrowth on day 84 and five imidacloprid-moxidectin-treated dogs had > 90% hair regrowth on day 84 (Fig. 5; Table 3).

**Table 2:** Number of dogs with skin lesions associated with generalized demodicosis

	Day −2	Day 28	Day 56	Day 84
	Fluralaner tablets			
Crusts	1/8 (12.5%)	1/8 (12.5%)	0/7 (0.0%)	0/7 (0.0%)
Scales	5/8 (62.5%)	1/8 (12.5%)	3/7 (42.9%)	0/7 (0.0%)
	Topical imidacloprid-moxidectin			
Crusts	2/8 (25.0%)	0/8 (0.0%)	0/8 (0.0%)	0/8 (0.0%)
Scales	7/8 (87.5%)	5/8 (62.5%)	6/8 (75%)	3/8 (37.5%)

**Table 3:** Assessment of hair regrowth on dogs before and after treatment for generalized demodicosis

Day of study	Score	Body areas with hair regrowth versus pre-administration assessment	Fluralaner tablets	Topical imidacloprid-moxidectin
	Assessment prior to any study treatments			
−2	1	Alopecia > 0 to 50% of the body	7/8 (87.5%)	7/8 (87.5%)
	2	Alopecia > 50 to ≤ 90% of the body	1/8 (12.5%)	1/8 (12.5%)
	3	Alopecia > 90% of the body	0/8 (0.0%)	0/8 (0.0%)
	Hair regrowth after treatment on days 0, 28, and 56^a^ and on day 84			
28	1	> 0 to 50% hair regrowth	2/8 (25.0%)	5/8 (62.5%)
	2	> 50 to ≤ 90% hair regrowth	4/8 (50.0%)	3/8 (37.5%)
	3	> 90% improvement	2/8 (25.0%)	0/8 (0.0%)
56	1	> 0 to 50%	0/7 (0.0%)	4/8 (50.0%)
	2	> 50 to ≤ 90%	2/7 (28.6%)	2/8 (25.0%)
	3	> 90%	5/7 (71.4%)	2/8 (25.0%)
84	1	> 0 to 50%	0/7 (0.0%)	1/8 (12.5%)
	2	> 50 to ≤ 90%	0/7 (0.0%)	2/8 (25.0%)
	3	> 90%	7/7 (100.0%)	5/8 (62.5%)

^a^Hair regrowth assessments were made before treatments were administered on days 28 and 56

## Discussion

Three monthly treatments with the flavored fluralaner 5.46% chewable tablets were effective in achieving parasitological cure in client-owned dogs with generalized demodicosis. No mites were found on any fluralaner-treated dog after the day 28 assessment, while up to 12 applications with topically applied imidacloprid-moxidectin achieved parasitological cure in two of eight dogs.

These findings are consistent with other reports from the same laboratory demonstrating the elimination of natural infestations with *D. canis* mites by different formulations of fluralaner and the variable efficacy of repeated treatments with imidacloprid-moxidectin. In the first report, mite counts were reduced by 99.8% on day 28 and by 100.0% on days 56 and 84 following a single oral fluralaner treatment [7]. In that study, following three monthly applications of imidacloprid-moxidectin to the control group, mite counts were reduced by 98.0%, 96.4%, and 94.7% on days 28, 56, and 84, respectively. Similarly, a 2019 report described just one of eight dogs as positive for *D. canis* mites 56 days following a single topical fluralaner treatment, and efficacy was 100.0% at the final assessment on day 84 [17]. Dogs in that study that received three monthly treatments with imidacloprid-moxidectin had mite count reductions of 9.8%, 45.4%, and 0.0% at days 28, 56, and 84, respectively.

Oral administration of other isoxazoline compounds has also been demonstrated to be effective in the treatment of generalized demodicosis using this laboratory model [8, 18, 19]. These laboratory results have been confirmed in field studies, although just two of those reported studies were well controlled (i.e., included a positive or negative control group). In one well-controlled study, 39 of 42 enrolled dogs were mite-free at the day 90 assessment following monthly treatments with sarolaner, compared with 17 of 22 dogs treated on multiple occasions with topical imidacloprid-moxidectin [8]. In the other study, 50/50 and 49/50 dogs receiving a single treatment with either the oral or topical fluralaner formulations, respectively, were free of mites on day 84, as were 21 of 24 dogs treated multiple times with imidacloprid-moxidectin [9]. Thus, the collective results of well-controlled studies indicate that the isoxazolines, particularly all three formulations of fluralaner, provide superior efficacy compared with imidacloprid-moxidectin for the treatment of canine generalized demodicosis.

Parasitological cure, based on two negative deep skin scrapings 1-month apart, does not guarantee a clinical cure, as shown in the current study by one imidacloprid-moxidectin-treated female that continued to exhibit severe clinical signs on day 84 despite zero mite counts on days 56 and 84. The absence of pyoderma from this dog’s biopsies on days −2 and 27 would appear to rule out a bacterial infection, particularly as cefovecin treatment was continued throughout the study. Other possible causes of the ongoing dermatosis would include an undiagnosed endocrinopathy or hereditary disorder [4].

Equally, a strong clinical response does not necessarily indicate parasitological cure: five imidacloprid-moxidectin-treated dogs improved from severe to mild clinical signs, despite counts of up to 31 mites continuing through day 84 (one dog that improved from severe to moderate had a Day 84 count of 65 mites). Even with both clinical and parasitological cure, it is important to remember the complex *D. canis* disease pathogenesis. Predisposing underlying health conditions including hypothyroidism and neoplasia, or immunosuppressive drug treatment are factors that can precipitate clinical demodicosis in adult dogs [4]. Therefore, relapse could occur in successfully treated dogs with continuing underlying health disorders.

The study demonstrates that three consecutive monthly doses of this oral formulation of fluralaner provide equivalent high efficacy in the treatment of generalized demodicosis as a single treatment with the original long-acting oral and the topically applied fluralaner formulations [7, 9, 17].

## Conclusion

This is the first study to demonstrate that three consecutive monthly doses of an orally administered flavored chewable fluralaner (5.46% w/w) tablet at a minimum dose of 10 mg/kg body weight can eliminate *D. canis* mites from dogs diagnosed with generalized demodicosis.

## Data Availability

Data from this study are proprietary and maintained by MSD Animal Health.
