# Correction to: Fluralaner 5.46% (w/w) favored chewable tablet (Bravecto® 1-Month) is effective for treatment of canine generalized demodicosis

**DOI:** 10.1186/s13071-022-05256-0

**Published:** 2022-04-08

**Authors:** Nadja Rohdich, Leon Meyer, Frank Guerino

**Affiliations:** 1 MSD Animal Health Innovation GmbH, Zur Propstei, 55270 Schwabenheim, Germany; 2 Clinvet International (Pty) Ltd, Uitzich Road, Bainsvlei, Bloemfountein, South Africa; 3 Merck Animal Health, 2 Giralda Farms, Madison, NJ 07940 USA

## Correction to: Parasites Vectors 15:83 (2022) https://doi.org/10.1186/s13071-022-05213-x

Following publication of the original article [1], it came to the authors’ attention that some incorrect data had been provided in Table 1. The published article has been updated with the corrected table, and this table may be seen below.

**Table 1:** Results from counts of *Demodex canis* mites obtained from deep skin scrapings

	Fluralaner tablets	Topical imidacloprid-moxidectin
Day − 2		
Arithmetic mean (SD)	639.9 (589.9)	608.5 (459.5)
Median	507.5	459.0
Range	19‒1764	90‒1314
Geometric mean	347.5	432.3
Day − 28		
Arithmetic mean (SD)	2.0 (2.8)	63.9 (118.3)
Median	0.0	17.0
Range	0‒6	0‒351
*P*-value (vs Day − 2)	0.0005*	< 0.0001*
Test Statistics	*t*_*(19)*_ = 4.18	*t*_*(21)*_ = 5.05
Geometric mean	1.0	17.2
Day − 56		
Arithmetic mean (SD)	0 (0.0)	34.1 (70.3)
Median	0.0	11.0
Range	0‒0	0‒207
*P*-value (vs Day − 2)	0.0007*	< 0.0001*
Test Statistics	*t*_*(19)*_ = 4.05	*t*_*(21)*_ = 5.33
Geometric mean	0.0	8.5
Day − 84		
Arithmetic mean (SD)	0 (0.0)	15.0 (22.8)
Median	0.0	3.5
Range	0‒0	0‒65
*P*-value (vs Day − 2)	0.0007*	< 0.0001*
Test statistics	*t*_*(19)*_ = 4.05	*t*_*(21)*_ = 5.51
Geometric mean	0.0	5.1

The authors thank you for reading and apologize for any inconvenience caused.
